# Using regression tree analysis to examine demographic and geographic characteristics of COVID-19 vaccination trends over time, United States, May 2021–April 2022, National Immunization Survey Adult COVID Module

**DOI:** 10.1016/j.vaccine.2024.126372

**Published:** 2024-10-04

**Authors:** Morgan Earp, Lu Meng, Carla L. Black, Rosalind J. Carter, Peng-Jun Lu, James A. Singleton, Terence Chorba

**Affiliations:** a U.S. Centers for Disease Control and Prevention, National Center for Health Statistics, United States; b U.S. Centers for Disease Control and Prevention, National Center for Emerging and Zoonotic Infectious Diseases, United States; c U.S. Centers for Disease Control and Prevention, National Center for Immunization and Respiratory Diseases, United States; d U.S. Centers for Disease Control and Prevention, National Center for HIV, Viral Hepatitis, STD, and TB Prevention, United States

**Keywords:** COVID-19 / prevention & control, Ethnicity, SARS-CoV-2, COVID-19 vaccines, COVID-19 vaccination, Regression tree, Machine learning

## Abstract

Using data from the nationally representative National Immunization Survey (NIS), we applied conditional linear regression tree methodology to examine relationships between demographic and geographic factors and propensity of receiving various doses of COVID-19 vaccine over time; these analyses identified temporal changes in these relationships that heretofore had not been identified using conventional logistical regression methodologies.

Three regression tree models were built using an R package, Recursive Partitioning for Modeling Survey (rpms), to examine propensities over time of receiving a (1) first dose of a two-dose COVID-19 mRNA primary vaccination series or single dose of the Janssen vaccine (vaccine initiation), (2) primary series completion, and (3) monovalent booster dose, using a conditional linear effect model. Persons ≥50 years were more likely to complete a primary series and receive a first booster dose; persons reporting having received non-COVID-19 vaccines recently were more likely to initiate vaccination, complete the primary series, and get a first booster dose; persons reporting having work or school requirements were more likely to complete the primary series. Persons not reporting having received non-COVID-19 vaccines in 2 years but reporting having work or school vaccination requirements were more likely to initiate vaccination than those without work/school requirements. Among persons not reporting having received non-COVID-19 vaccines in 2 years and not reporting having work or school vaccination requirements, those aged ≥50 years were more likely to initiate vaccination than were younger adults. Propensity of receiving various doses was correlated with age, having recently received non-COVID 19 vaccines, and having vaccination requirements at work or school.

Regression tree methodology enabled modeling of different COVID-19 vaccination dose propensities as a linear effect of time, revealed changes in relationships over time between demographic factors and propensity of receipt of different doses, and identified populations that may benefit from vaccination outreach efforts.

## 1. Introduction

In August–November 2021, the U.S. Food and Drug Administration approved several Emergency Use Authorizations (EUAs) for COVID-19 monovalent booster vaccinations, including an EUA for an additional primary dose for immunocompromised persons to achieve primary-dose completion and an EUA for a booster dose for all adults aged ≥18 years after ≥2 months following vaccination with the one-dose Johnson & Johnson/Janssen (J&J) primary series or after ≥5 months following the second dose of the Pfizer-BioNTech or Moderna two-dose mRNA primary series [1]. Using logistic regression models, disparities in COVID-19 vaccine booster uptake have been shown to be related to socioeconomic status, insurance status, disability, and social demographic factors, including age, education level, race/ethnicity, and residency in rural or urban areas [2–5]. In October 2022, the Centers for Disease Control and Prevention (CDC) published a study in which cloud-based machine learning methods were used to form a classification tree algorithm to identify and describe relationships and interactions of demographic factors associated with the receipt or non-receipt of a COVID-19 booster vaccine among eligible persons aged ≥18 years in the U.S. [6]. The model that was generated identified groups less likely to be boosted being persons aged 18–34 years, recipients of the Johnson & Johnson primary series, persons from racial/ethnic minority groups, residents of non-large metro areas, and those living in communities in the South with high CDC/ATSDR Social Vulnerability Index (SVI) scores. Factors included in SVI scores include socioeconomic status, household composition, disability, minority status, housing type, and transportation [7]. A lower SVI score for any given individual would mean that the population in that person’s county of residence was less socially vulnerable, i.e., the population lives/works in settings that put that population at decreased risk of exposure to hazards and increased access to health care resources. While several published studies have identified risk factors for nonreceipt of COVID-19 vaccines [2–6,8], none has examined trends over time in the relationships between these factors and vaccine uptake.

Since April 2021, CDC has conducted the National Immunization Survey Adult COVID Module (NIS-ACM) among persons aged ≥18 years to monitor COVID-19 vaccine uptake and confidence in vaccination [8]. In the present study, we applied conditional linear regression tree methodology to NIS-ACM data using recursive partitioning to examine the relationships over time between demographic and geographic factors and the propensity of COVID-19 vaccine initiation (receiving a first dose), primary series completion, and a monovalent booster dose completion. We analyzed data from the NIS-ACM using an R regression tree package, Recursive Partitioning for Modeling Survey (rpms) [9], creating regression tree models using a conditional linear model fit on time in year for each node of the tree. Regression trees offer several advantages over more traditional logistical regression, and rpms offers additional advantages over other regression tree models. Regression trees (1) can automatically identify associated variables and their optimal splitting or categorical groupings; (2) automatically detect statistically significant interaction effects; (3) use missing data as a valid input into the model for categorical variables; (4) are nonparametric and can model nonlinear effects; and (5) can be used to easily identify and describe mutually exclusive subgroups with varying levels of an outcome [10–12]. Specifically, rpms can also: (1) automatically detect statistically significant differences in intercepts and slopes, not just means and proportions; (2) produce visualizations that allow the user to easily identify and describe the relationship between the outcome of interest and input factors with mutually exclusive subgroups over a linear continuum, not just a single point in time; (3) provide unbiased variable selection and stable propensity score estimates using randomized permutation testing; and (4) adjust for complex sample design and produce-consistent coefficients [10–12]. One other specific advantage of using rpms when examining vaccination coverage is that rpms allows one to examine if demographic and geographic subgroups have varying coverage propensities over time, and this study successfully demonstrated this advantage and others enumerated above.

## 2. Methods

### 2.1. Data

The NIS-ACM uses the random digit dialed cellular telephone sample of the National Immunization Survey (NIS) to survey adults in the United States, stratified by state and selected local areas and U.S.-affiliated jurisdictions [13]. The NIS is used to monitor vaccination coverage, including routine child and teen vaccinations, as well as influenza and COVID-19 vaccinations. The NIS-ACM collects demographic and immunization data on all eligible persons ≥18 years. This paper focuses on modeling COVID-19 vaccine uptake propensity of US adults aged ≥18 years using 12 months of NIS-ACM data collected from April 22, 2021, to April 30, 2022. NIS-ACM data are split into approximate monthly data files for weighting and analytic use. Monthly sample sizes for the NIS-ACM ranged from 39,316 to 79,189 with an average of 65,536 per month. Survey weights were also calibrated by age and sex to state-level vaccine administration data reported to CDC by jurisdictions as of the middle of the monthly data collection period [14]. Response rates for the monthly datasets ranged from 17.2 % to 22.1 %.

This activity was reviewed from an ethics perspective and approved by CDC, deemed not research, and was conducted consistent with applicable federal law, ethics, and CDC policy (45C.F.R. part 46.102(l) (2), 21C.F.R. part 56; 42 U.S.C. §241(d); 5 U.S.C. §552a; 44 U.S.C. §3501). The activities that generated the NIS data set on which these secondary analyses were performed had been approved by the Centers for Disease Control and Prevention and the National Opinion Research Center at the University of Chicago Institutional Review Board. The NIS-ACM respondents provide verbal consent to participate. Personal identifiable information is not collected from the survey participants.

### 2.2. Analysis

Using the R regression tree package rpms [15], we modeled COVID-19 vaccine initiation (receipt of first dose of the two-dose Pfizer-BioNTech or Moderna COVID-19 vaccine series or a single dose of the Janssen COVID-19 vaccine (Johnson & Johnson)), primary series completion (completing two-dose Pfizer-BioNTech or Moderna COVID-19 vaccine series or having received the single dose of the Janssen COVID-19 vaccine), and first monovalent booster dose completion propensities as a linear effect of time in months using the NIS-ACM monthly data. We built three regression trees using rpms: 1) modeling the cumulative propensity of COVID-19 vaccine initiation and the linear relationship over time of vaccine uptake controlling for demographic and geographic characteristics during May 2021 (time = 0) through April 2022 (time = 11); 2) modeling both the linear relationship over time controlling for demographic and geographic characteristics and the propensity of primary series completion from May 2021 (time = 0) through April 2022 (time = 11); and 3) modeling both the linear relationship over time controlling for demographic and geographic characteristics and the propensity of first booster dose completion (from October 2021 (time = 0) through April 2022 (time = 6).

The following demographic and geographic variables were controlled for in all models: age, sex, race/ethnicity, income/poverty, reporting having received a non-COVID 19 vaccine in the past 2 years, health insurance status, education, essential worker status, Department of Health and Human Services region, receiving a health care worker recommendation for a vaccine, work/school requirement, metropolitan statistical area (MSA), reporting previously having had COVID-19, and SVI of county of residence.

All input variables were categorical variables. Missingness (e.g., not reported) within categorical variables can be treated as valid values in the model using the rpms package, which minimizes the loss of observations due to data incompleteness. The first tree model was built based on all 786,429 observations (unique persons) that had no missing value on first dose of the COVID-19 vaccine. The second tree model was built based on the 669,455 persons (of the 786,429 who initiated receiving a COVID-19 vaccine) with complete data on completion of the primary series of COVID-19 vaccine. The third tree model was built based on 365,504 out of 443,743 observations (who had completed the primary series) with complete data on first booster completion, beginning in October 2021. Table 1 presents the detailed monthly sample size and breakdown by COVID-19 vaccine initiation, primary series completion, and first booster dose completion.

All trees were constructed using a *p*-value cutoff of 0.0001 and a minimum bin size (end node size) equivalent to 10 % [16] of the dose completion dataset; COVID vaccine initiation model minimum bin size = 78,643; primary series completion model minimum bin size = 66,946; and first booster dose model minimum bin size = 36,550. The rpms algorithm uses a permutation test described in Toth (2020) to determine if each split is significant. Once the algorithm fails to find a significant split given the *p*-value cutoff and minimum bin size, the tree stops splitting. The subgroups identified at each split are referred to as regression tree nodes. The mutually exclusive subgroups identified at the bottom of the tree are referred to as end nodes or leaves. The first node includes the entire data set before splitting and is referred to as the root node and is labeled Node 1. The first left child node of the root node is labeled Node 2 and the right child node of the root node is labeled Node 3. This leads to every node getting a unique node label.

## 3. Results

### 3.1. Overview

**Fig. 1.**
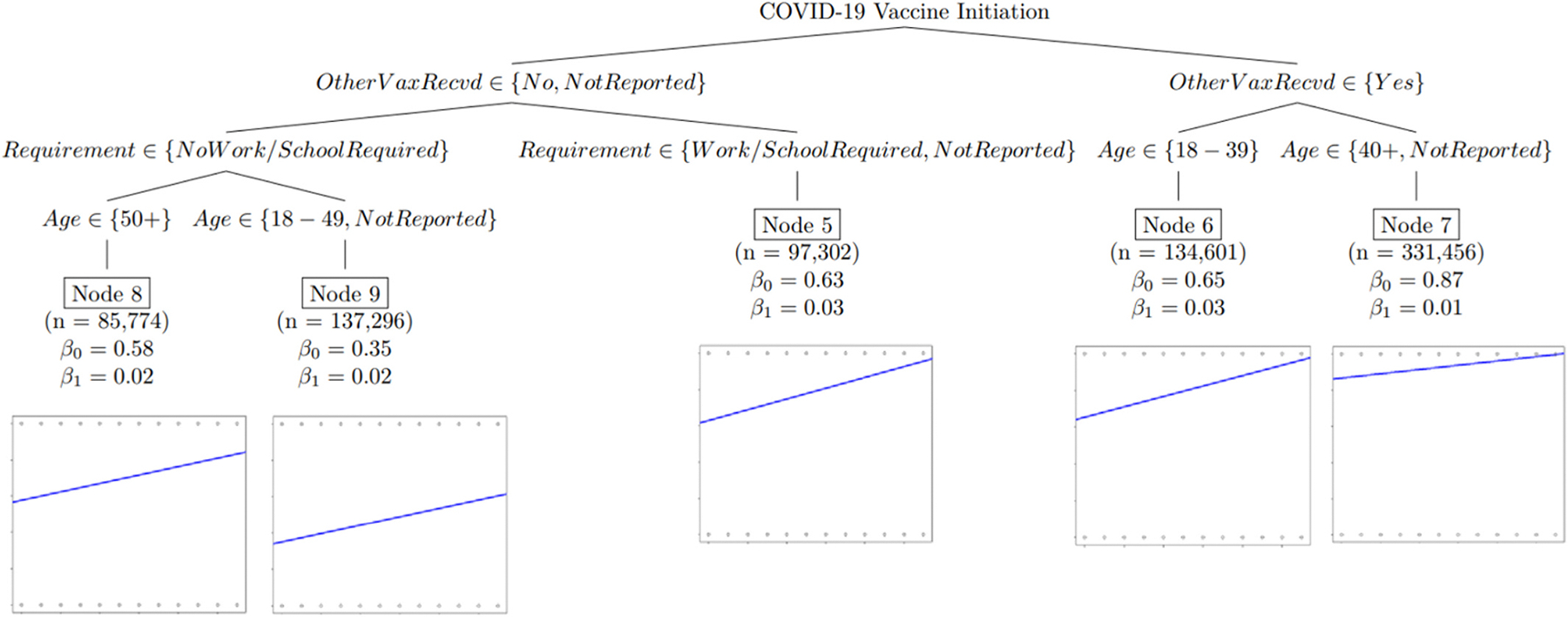
Model One - Linear regression tree model assessing the linear effect of time in months on COVID-19 vaccine initiation propensities, controlling for demographic and geographic characteristics.

**Fig. 2.**
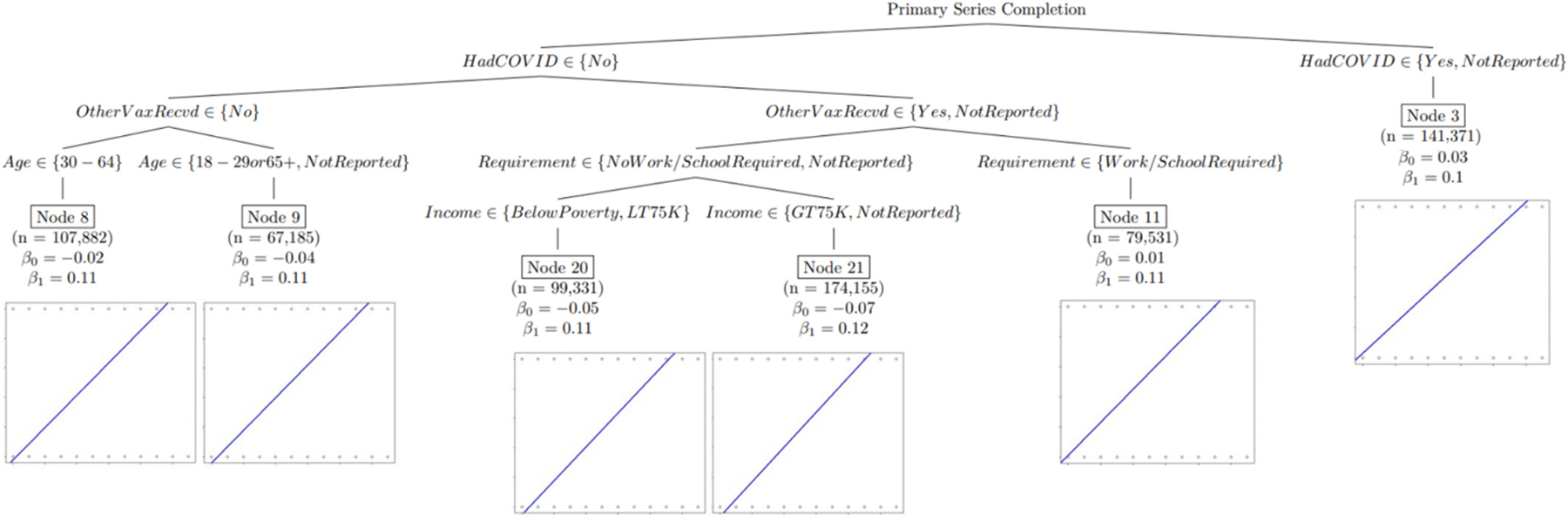
Model Two - Linear regression tree model assessing the linear effect of time in months on COVID-19 primary series completion propensities, controlling for demographic and geographic characteristics*. * Negative intercepts for some groups are due to time lag and varies by state when the vaccine had been rolled out to less vulnerable groups. Abbreviations: LT75K–annual household income less than $75 k but above poverty threshold GT75K–annual household income greater than $75 k

**Fig. 3.**
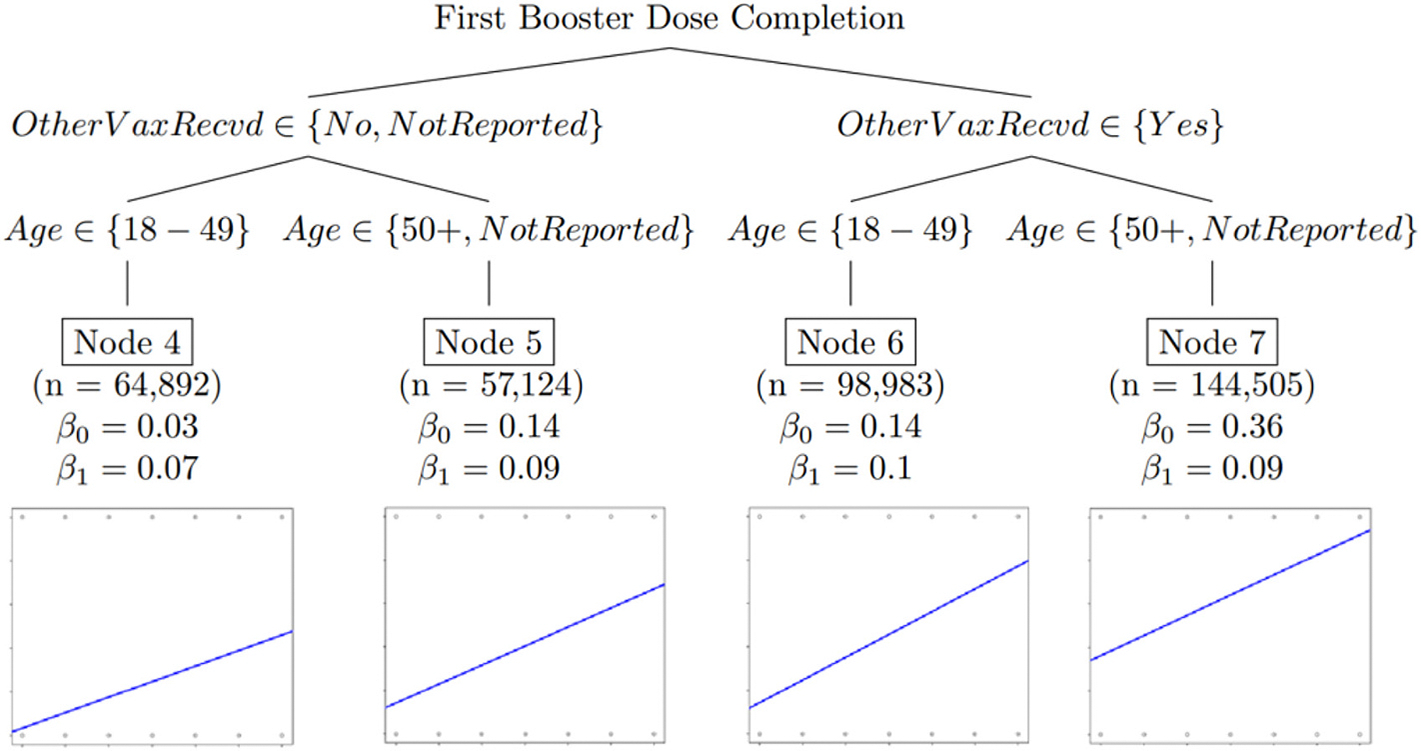
Model Three - Linear regression tree model assessing the linear effect of time in months on COVID-19 first booster dose completion propensities, controlling for demographic and geographic characteristics.

In the first of the two regression tree models described above, shown in Fig. 1 below, the linear effect of time on COVID-19 vaccine initiation propensity is presented. The second regression tree, shown in Fig. 2 below, presents the linear effect of time on COVID-19 primary series completion propensity, and the third regression tree, shown in Fig. 3, presents the linear effect of time on COVID-19 first booster dose propensity. All three regression tree models control for demographic and geographic characteristics. The y-intercept (β_0_) represents the average reported vaccination rate for respondents at time zero, and the slope (β_1_) is the effect on reported vaccination rate for every additional month. Table 2 presents an overview of the demographic and geographic characteristics included in the model, and the significant demographic and geographic characteristic relationships in Models One, Two, and Three.

### 3.2. Model One – COVID-19 Vaccine Initiation Propensities Over Time

The first model indicated that persons who reported having received non-COVID-19 vaccines in the past 2 years (see Fig. 1 first split to the right, Nodes 6 & 7, the cumulative intercept *β*_*0*_ = 0.81) were more likely to report having received a COVID-19 first dose (by about 31 percentage points), than those not having received a non-COVID-19 vaccine in the past 2 years or those who did not report their vaccination status (see Fig. 1 first split to the left, Nodes 5, 8, & 9, the cumulative intercept *β*_*0*_ = 0.50). Persons who reported having received non-COVID-19 vaccines in the past 2 years aged ≥40 years or persons not reporting age were most likely to report having received aCOVID-19 first doseinitially with a one percentage point increase over time (see Fig. 1, Node 7, *β*_*1*_═0.01).; and those aged 18–39 years saw a steady increase of about 3 percentage points every month (*β*_*1*_═0.03) over 11 months, taking them from an initial first dose coverage of 65 % (*β*_*0*_ = 0.65*)* to 98 % (see Fig. 1, Node 6).

For those not having received a non-COVID-19 vaccine in the past 2 years (or not reporting their vaccination status), those reporting not having a school or work requirement were less likely to report being vaccinated, especially those aged 18–49 years (or not reporting age) (see Fig. 1, Node 9). Those aged ≥50 years not having received a non-COVID-19 vaccine in the past two years (or not reporting their vaccination status) and reporting not having a school or work requirement had a higher first dose COVID-19 vaccine initiation propensity than those aged 18–49 years (or those not reporting age) (see Fig. 1, Node 8 & 9, *β*_*0*_). Those not reporting the vaccination status or reporting not having received a non-COVID-19 vaccine in the past 2 years and reporting a work or school requirement (or not reporting whether they had a work/school requirement) saw first dose COVID-19 vaccine initiation propensities and increases over time like those for adults aged18 to 39 years who reported having received non-COVID-19 vaccines in the past 2 years (see Fig. 1, Nodes 5 & 6, *β*_*0*_ &*β*_*1*_).

### 3.3. Model Two – Primary Series Completion Propensities Over Time

Due to the recommended timing lag (at least 21 days between first and second primary dose of Pfizer COVID-19 vaccine and 28 days between first and second primary dose of Moderna COVID-19 vaccine) between first dose and primary series completion, model intercepts (*β*_*0*_) in the second model have negative initial starting values for some groups, since both the first and second model use the same initial start month of April 2021 (Fig. 2). The second model indicated that persons who reported having had COVID-19 or did not report whether they previously had COVID-19 were initially more likely to report having completed the COVID-19 primary series (highest initial percentage of reported series completion *β*_*0*_ = 0.03), but were slightly less likely over time to report having completed the COVID-19 primary series (lowest increasing percentage point *β*_*1*_ = 0.10), compared with persons who reported not having had COVID-19.

Among those reporting not having previously had COVID-19, having received non-COVID-19 vaccines (or declining to report), and having no school or work COVID-19 vaccination requirement (or declining to report), those who reported having income level greater than $75,000 (or not reporting income) were the least likely to complete the COVID-19 primary series initially (lowest initial percentage *β*_*0*_ = −0.07), but over time had the steepest positive trend for completing the COVID-19 primary series (highest increasing percentage point *β*_*1*_ = 0.12), compared with other persons.

Among those who reported never having had COVID-19 and having received non-COVID-19 vaccines (or declining to report), those who reported having had a work or school requirement were more likely to report having completed the primary dose initially (*β*_*0*_ = 0.01), compared with all other groups who never had COVID-19.

### 3.4. Model Three – First Booster Dose Completion Propensities Over Time

The third model indicated that older persons had higher first booster doses initially and higher uptake over time, with those aged ≥50 years having the highest initial first dose propensity and a nine-percentage point increase in propensity every month (See Fig. 3, Node 5, *β*_*1*_ = 0.09).

Those aged ≥50 years (or not reporting age) who reported having received non-COVID-19 vaccines in the past 2 years were more likely to report having received a COVID-19 booster dose initially (by about 22 percentage points), compared with those aged 18–49 years (see Fig. 3, Nodes 6 & 7, *β*_*0*_). Those who did not report or reported not having received non-COVID-19 vaccines in the past 2 years_,_ ages ≥50 years (or not reporting age) were more likely to report having received a COVID-19 booster dose initially (by about 11 percentage points), compared with those aged 18–49 years (see Fig. 3, Nodes 4 & 5, *β*_*0*_).

## 4. Discussion

Our analysis of data from the NIS-ACM using an R regression tree package yielded *both* a substantive perspective on the relationships over time between demographic and geographic factors and the propensity of COVID-19 vaccine initiation (receiving a first dose), completion of primary series, or a monovalent booster dose of the COVID-19 vaccine *and* lessons in using a novel approach to modeling the COVID-19 vaccine initiation, primary series completion, and first booster dose propensities as a linear effect of time in months while easily describing subgroups with higher or lower propensities. Use of the NIS-ACM provided a rich set of demographic input variables from a rigorous survey with weights and design intended to be nationally representative, unlike heretofore-published logistic regression models that may not have been generalizable to the U.S. population because they were based on data from other populations or lacked data for specific variables, were unable to capture complex interactions, or lacked visualization of trends over time in the relationships between demographic factors and vaccination propensities [2–6]. In describing the benefits of this approach, our discussion has two areas of focus: 1) analytic insights about COVID-19 vaccination, and 2) methodologic considerations, i.e., what we learned in working with the rpms as a methodology and what was unique in our use of the technology:

### 4.1. Analytic summary

In our analyses, persons reporting having received a non-COVID-19 vaccine in the past 2 years were more likely to initiate receiving a COVID-19 vaccine than those not reporting having received a non-COVID 19 vaccine. This is consistent with findings from other surveys on adult vaccination assessment that have indicated that adults who obtained other recommended vaccines, e.g., human papillomavirus (HPV) or annual influenza vaccine, were more likely to obtain COVID-19 vaccination [17–19]. As has been reported in other settings in which making employment contingent upon vaccination against influenza has resulted in dramatic uptake of influenza vaccination, we found that persons not reporting having received non-COVID-19 vaccines in the past 2 years but reporting having a work or school vaccination requirement were more likely to initiate receiving a COVID-19 vaccine than those without a work/school requirement [20–21]. Similarly, among persons not reporting having received non-COVID-19 vaccines in the past 2 years and not reporting having a work or school vaccination requirement, those who were aged ≥50 years were more likely to initiate receiving a COVID-19 vaccine than younger adults. This finding reinforced previous findings in the literature that identified older age as a common predictor of COVID-19 vaccine uptake [22–23].

Persons aged ≥50 years were most likely to get a first booster dose. This was consistent with CDC recommendations at that time that persons who received their primary series who were aged ≥65 and persons who resided in long term care settings should receive a COVID-19 booster ≥6 months after completing their primary vaccination series [24]. Persons aged 18–49 years reporting having received non-COVID-19 vaccines in the past 2 years were more likely to get a first booster dose than those not reporting having received a non-COVID-19 vaccine. Among persons who reported having received non-COVID-19 vaccine in the past 2 years, those who were aged ≥50 years were more likely to get a first booster dose than those aged 18–49 years.

Overall, the models demonstrated that: 1) older adults were more likely to initiate receiving a COVID-19 vaccine, complete their primary series (among those not previously having had COVID-19 and not having received any non-COVID-19 vaccinations in the previous 2 years), and get a first booster dose than younger persons; 2) persons reporting having received a non-COVID-19 vaccine in the past 2 years were more likely to initiate receiving a COVID-19 vaccine, complete their primary series dose (among those not reporting previously having COVID-19), and get a first booster dose than those not reporting having received a non-COVID-19 vaccine in the past 2 years; and 3) persons reporting having work or school COVID-19 vaccination requirements (or refusing to report work or school requirements) were more likely to initiate receiving a COVID-19 vaccine (conditional on not receiving or not reporting having received a non-COVID-19 vaccinations in the previous 2 years) and complete their primary series dose, given reporting not having previously had COVID-19 and reporting having had a previous non-COVID-19 vaccination in the previous 2 years (or refusing to report), than those reporting no work or school requirements.

### 4.2. Methodological considerations

From a methodological perspective, the choice of the R regression tree package rpms was made for its advantages as an approach to modeling the COVID-19 vaccine initiation, primary dose completion, and first booster dose propensities as a linear effect of time in months using the NIS-ACM monthly data. While we could have used logistic regression to model vaccination propensities, regression trees are able to handle missing data automatically and are able to identify moderating and interaction effects and can be used to easily describe subgroups with higher or lower propensities [10,11,24–29]. Unlike most prior studies using logistic regression models, using rpms enabled the examination of trends over time in the relationships between demographic and geographic factors and vaccination propensities, e.g., among persons having received a recent non COVID-19 vaccination, those aged 18 to 39 years were less likely to initiate receiving a COVID-19 vaccine compared with persons 40 years or older in April 2021 (the beginning of the study period), but over time had a steeper positive trend for vaccine initiation; similarly, persons with income >$75,000 were least likely to complete the COVID-19 primary series at the beginning of the study period, but over time had the steepest positive trend for completion, compared with persons in other income categories.

In sum, the rpms package can be used to identify key group differences both in terms of means and in terms of intercepts and slopes when a linear term (e.g., time) is added to the model. The rpms regression tree package recursively partitions the data, fitting a specified linear model on each node separately. The rpms recursive partitioning algorithm splits variables and selects splits using a randomized permutation test procedure that provides unbiased variable selection, adjusts for complex sample design, and produces design-consistent coefficients [30].

### 4.3. Limitations

Five limitations might be considered when interpreting these findings. First, NIS-ACM has a low response rate (<23 %). As noted in NIS-ACM reports elsewhere, although survey weights were calibrated to COVID-19 vaccine administration data to mitigate at least some of the possible bias from incomplete sample frame (for example, sampling did not cover the landline-only and phoneless populations), nonresponse, and misclassification of vaccination status, bias estimates might remain after weighting [31]. Second, COVID-19 vaccination was self-reported and might be subject to recall or social desirability bias. Third, the NIS-ACM sampled noninstitutionalized adults via cellular telephone; therefore, adults who were nursing home residents or were incarcerated might not have been represented. Fourth, because these findings rely on self-reported data, survey estimates of COVID-19 vaccination coverage might differ from reported vaccine administration data and other data reported at https://covid.cdc.gov/covid-data. Fifth, the findings of this analysis are based on predictive modeling, and do not take into account the complex causal pathways that lead to vaccination decisions by individuals. All the categories of the variables or options for each survey items included in the model were predetermined by the NIS-ACM survey. In addition, due to potential multicollinearity, there may have been a common set of demographic factors (such as race, education level, geographic location/residency) associated with receipt of COVID-19 in our data that were not identified as significant factors in the current tree model [32–33]. In the supplemental tables A-1 to A-3, we have displayed details on the nodes/splits of each tree model by all factors included in the present study.

## 5. Conclusions

We applied an R regression tree package to monthly data from the NIS-ACM to perform recursive partitioning, creating decision trees using a linear model fit on time in survey for each node, to examine the relationships over time between demographic and geographic factors and the propensity of COVID-19 vaccine initiation, primary series completion, or receiving a booster dose of the COVID-19 vaccine. In general, persons aged ≥50 years were more likely to initiate receiving a COVID-19 vaccine and to get a first booster dose than younger persons; persons reporting having received a non-COVID 19 vaccine in the past 2 years were more likely to initiate receiving a COVID-19 vaccine, complete their primary series, and get a first booster dose than those not reporting having received a non-COVID-19 vaccine in the past 2 years; persons reporting having work or school requirements were more likely to initiate receiving a COVID-19 vaccine and complete their primary series than those reporting no work or school requirements. The choice of the R regression tree package rpms was favored over logistic regression for modeling the different dose propensities as a linear effect of time in months using the NIS-ACM data.

## Supplementary Material

Supplemental Tables : A-1, A-2, and A-3

## Figures and Tables

**Table 1:** Monthly sample size and breakdown by COVID-19 vaccine initiation, primary series completion, and first booster dose completion.

Month	Data collection period	Total sample size (*n* = 786,429)	COVID-19 Vaccine Initiation (*n* = 786,429)		Primary Series Completion (*n* = 669,455)		First Booster Dose Completion (*n* = 365,504)	
			Yes	No	Yes	No	Yes	No

2021 May	Apr 22-May 29, 2021	77,006	58,171	18,835	3392	54,779	NA	NA
2021 Jun	May 30-Jun 26, 2021	56,567	45,477	11,090	2967	42,510	NA	NA
2021 Jul	Jun 27-Jul 31, 2021	73,181	60,652	12,529	4102	56,550	NA	NA
2021 Aug	Aug 1-28, 2021	62,874	52,971	9903	3466	49,505	NA	NA
2021 Sep	Aug 29-Sep 25, 2021	73,058	62,847	10,211	4311	58,536	NA	NA
2021 Oct	Sep 26-Oct 30, 2021	79,189	68,368	10,821	52,466	15,902	5781	46,685
2021 Nov	Oct 31-Nov 27, 2021	39,316	34,335	4981	33,171	1164	9433	23,738
2021 Dec	Nov 28-Dec 31, 2021	68,343	60,745	7598	59,175	1570	29,177	29,998
2022 Jan	Jan 2-29, 2022	62,426	55,189	7237	53,850	1339	32,971	20,879
2022 Feb	Jan 30-Feb 26, 2022	58,256	51,041	7215	49,895	1146	33,085	16,810
2022 Mar	Feb 27-Mar 26, 2022	62,829	55,218	7611	54,022	1196	37,232	16,790
2022 Apr	Mar 27-Apr 30, 2022	73,384	64,441	8943	62,925	1516	43,315	19,610

**Table 2:** Demographic and geographic characteristics related to COVID-19 vaccine initiation, primary dose completion, and first booster dose propensities over time, and significant demographic and geographic characteristic relationships in Models One, Two, and Three.

Control Variables Description	Short Name in Figure	Categories	Model 1: COVID-19 Vaccine Initiation	Model 2: Primary Series Completion	Model 3: First Booster Dose Completion
Age (years)	Age	▪ 18-29 ▪ 30-39 ▪ 40-49 ▪ 50-64 ▪ 65-74 ▪ 75+ ▪ Not reported	Ages 18 to 49 or Not-reported versus 50+, given Other vaccine received = No or Not-reported, and Work/School requirement = No Ages 18 to 39 versus 40+ or Not reported, given Other vaccine received = Yes	Ages 30-64 versus 18-29, 65+, or Not reported, given Previously had COVID = No and Other vaccine received = No	Ages 18 to 49 versus 50+ or Not reported, given Other vaccine received = No or Not reported Ages 18 to 49 versus 50+ or Not reported, given Other vaccine received = Yes
Race/Ethnicity	-	▪ Hispanic ▪ Non-Hispanic (NH) American Indian /Alaskan Native ▪ NH Asian ▪ NH Black ▪ NH Pacific Islander/Native Hawaiian ▪ NH White ▪ NH Multiple Race ▪ Not reported	Not significant	Not significant	Not significant
Sex	-	▪ Female ▪ Male ▪ Not reported	Not significant	Not significant	Not significant
Income (U.S. dollars)	Income	▪ Below poverty ▪ Less than (LT) 75 K but above poverty ▪ Greater than (GT) 75 K ▪ Not reported	Not significant	Below poverty or less than 75 k versus Greater than 75 k or Not reported, given Work/School requirement = No or Not reported, and Other vaccine received = Yes or Not reported, and Previously had COVID = No	Not significant
Education	-	▪ Greater than (GT) high school ▪ Less or equal (LTE) to high school ▪ Not reported	Not significant	Not significant	Not significant
Insurance	-	▪ Not insured ▪ Insured ▪ Not reported	Not significant	Not significant	Not significant
Essential Worker Status	-	▪ Healthcare ▪ School or childcare ▪ Other frontline workers ▪ Other essential workers ▪ Not essential workers ▪ Not reported	Not significant	Not significant	Not significant
Health Care Provider recommends vaccination	-	▪ No ▪ Yes ▪ Not reported	Not significant	Not significant	Not significant
Work/School requirement	Requirement	▪ No work/school required ▪ Work/school required ▪ Not reported	No Work/School requirement versus Work/School requirement or Not reported, given Other vaccine received = No or Not reported	No Work/School Requirement or Not Reported versus Work/School requirement, given Other vaccine received = Yes or Not reported, and Previously had COVID = No	Not significant
HHS Region*	-	HHS Region 1-10	Not significant	Not significant	Not significant
Metropolitan Statistical Area (MSA) Status	-	▪ MSA, Principal city ▪ MSA, Non-principal city ▪ Non-MSA	Not significant	Not significant	Not significant
Social Vulnerability Index (SVI)	-	▪ Low ▪ Medium ▪ High ▪ Not reported	Not significant	Not significant	Not significant
Previously had COVID	HadCOVID	▪ No ▪ Yes ▪ Not reported	Not significant	Had COVID or Not reported versus Not had COVID	Not significant
Other vaccine received (received non-COVID-19 vaccines inthe past 2 years)	OtherVaxRecvd	▪ No ▪ Yes ▪ Not reported	No or Not reported versus Yes	Yes or Not reported versus No, given Previously had COVID = No	No or Not reported versus Yes

* HHS Regional Offices | HHS.gov

## Data Availability

The authors do not have permission to share data.

## References

[1] Centers for Disease Control and Prevention. COVID-19 ACIP Vaccine Recommendations. ACIP Recommendations: COVID-19 Vaccine | ACIP Recommendations | CDCACIP Recommendations: COVID-19 Vaccine | ACIP Recommendations | CDC. Available from, https://www.cdc.gov/vaccines/hcp/acip-recs/vacc-specific/covid-19.html. [Accessed 8 September 2024].

[2] Bendezu-Quispe G, Caira-Chuquineyra B, Fernandez-Guzman D, UrrunagaPastor D, Herrera-Añazco P, Benites-Zapata VA. Factors associated with not receiving a booster dose of COVID-19 vaccine in Peru. Vaccines (Basel) 2022;10(8):1183. 10.3390/vaccines10081183. 35893832 PMC9330573

[3] Yoshida M, Kobashi Y, Kawamura T, Shimazu Y, Nishikawa Y, Omata F, Factors associated with COVID-19 vaccine booster hesitancy: a retrospective cohort study, Fukushima vaccination community survey. Vaccines (Basel) 2022;10(4):515. 10.3390/vaccines10040515. 35455264 PMC9032295

[4] Lee RC, Hu H, Kawaguchi ES, Soto DW, Shanker K, Klausner JD, COVID-19 booster vaccine attitudes and behaviors among university students and staff in the United States: the USC Trojan pandemic research initiative. Prev Med Rep 2022;28:101866. 10.1016/j.pmedr.2022.101866. 35785408 PMC9235287

[5] Gaffney A, Himmelstein DU, McCormick D, Woolhandler S. Disparities in COVID-19 vaccine booster uptake in the USA: December 2021–February 2022. J Gen Intern Med 2022;24:1–4. 10.1007/s11606-022-07648-5 PMC9128769 35610470

[6] Meng L, Fast HE, Saelee R, Zell E, Murthy BP, Murthy NC, Using a cloud-based machine learning classification tree analysis to understand the demographic characteristics associated with COVID-19 booster vaccination among adults in the United States. Open forum. Infect Dis Ther 2022;9(9):ofac446. 10.1093/ofid/ofac446. PMC9452182 36131845

[7] Ingram DD, Franco SJ. 2013 NCHS urban–rural classification scheme for counties. National Center for Health Statistics. Vital Health Stat 2014;2(166):1–73. 24776070

[8] Kriss JL, Hung MC, Srivastav A, Black CL, Lindley MC, Lee JT, COVID-19 vaccination coverage, by race and ethnicity - National Immunization Survey Adult COVID module, United States, December 2020-November 2021. MMWR Morb Mortal Wkly Rep 2022;71(23):757–63. 35679179 10.15585/mmwr.mm7123a2 PMC9181054

[9] Toth D Rpms: recursive partitioning for modeling survey data. R package version 0.2.0. Available from. https://CRAN.R-project.org/package=rpms. [Accessed 8 September 2024].

[10] Earp M, Kaplan R, Toth D. Modeling the relationship between proxy measures of respondent burden and survey response rates in a household panel survey. J Off Stat 2022;37(4):1145–75. 10.2478/jos-2022-0049. PMC11130710 38807963

[11] Earp M, Toth D, Phipps P, Oslund C. Assessing nonresponse in a longitudinal establishment survey using regression trees. J Off Stat 2018;34(2):463–81. 10.2478/JOS-2018-0021.

[12] Yang DK, Toth DS. Analyzing the association of objective burden measures to perceived burden with regression trees. J Off Stat 2022;38(4):1125–44.

[13] Wolter KM, Smith PJ, Khare M, Statistical methodology of the National Immunization Survey, 2005–2014. National Center for Health Statistics. Vital Health Stat 2017;1(61). Available from: https://www.cdc.gov/nchs/data/series/sr_01/sr01_061.pdf. 29466229

[14] Centers for Disease Control and Prevention. COVID Data Tracker. Available from. https://covid.cdc.gov/covid-data-tracker/#datatracker-home; 2024.

[15] Toth D Recursive partitioning for modeling survey data. R package version 0.4.0. Available from. https://CRAN.R-project.org/package=rpms; 2019.

[16] Everson TM, Niedzwiecki MM, Toth D, Tellez-Plaza M, Liu H, Barr DB, Metal biomarker mixtures and blood pressure in the United States: cross-sectional findings from the 1999–2006 National Health and nutrition examination survey (NHANES). Environ Health 2021;20(1):1–11. 33583418 10.1186/s12940-021-00695-1 PMC7883578

[17] Berenson AB, Chang M, Hirth JM, Kanukurthy M. Intent to get vaccinated against COVID-19 among reproductive-aged women in Texas. Hum Vaccin Immunother 2021;17(9):2914–8. 10.1080/21645515.2021.1918994. 34081572 PMC8381790

[18] Gatwood J, Shuvo S, Hohmeier KC, Hagemann T, Chiu CY, Tong R, Pneumococcal vaccination in older adults: an initial analysis of social determinants of health and vaccine uptake. Vaccine 2020;38(35):5607–17. 10.1016/j.vaccine.2020.06.077. 32654903

[19] Sherman SM, Sim J, Amlôt R, Cutts M, Dasch H, Rubin GJ, Intention to have the seasonal influenza vaccination during the COVID-19 pandemic among eligible adults in the UK: a cross-sectional survey. BMJ Open 2021;11(7):e049369. 10.1136/bmjopen-2021-049369. PMC8282414 34257095

[20] Pitts SI, Maruthur NM, Millar KR, Perl TM, Segal J. A systematic review of mandatory influenza vaccination in healthcare personnel. Am J Prev Med 2014;47(3):330–40. 25145618 10.1016/j.amepre.2014.05.035

[21] Rubenstein BL, Amiel PJ, Ternier A, Helmy H, Lim S, Chokshi DA, Increases in COVID-19 vaccination among NYC municipal employees after implementation of vaccination requirements: study examines COVID-19 vaccination rates among New York City municipal employees after vaccinations requirements were implemented. Health Aff 2023;42(3):357–65. 10.1377/hlthaff.2022.00809 PMC10917388 36877900

[22] Liang CK, Lee WJ, Peng LN, Meng LC, Hsiao FY, Chen LK. COVID-19 vaccines in older adults: challenges in vaccine development and policy making. Clin Geriatr Med 2022;38(3):605–20. 10.1016/j.cger.2022.03.006. 35868676 PMC8934735

[23] Levenson E Why older people are so much more vaccinated than younger people. CNN 2021. Available from, https://www.cnn.com/2021/08/28/us/older-adults-covid-vaccine/index.html.

[24] Mbaeyi S, Oliver SE, Collins JP, Godfrey M, Goswami ND, Hadler SC, The advisory committee on immunization practices’ interim recommendations for additional primary and booster doses of COVID-19 vaccines — United States, 2021. Morb Mortal Wkly Rep 2021;70:1545–52. 10.15585/mmwr.mm7044e2. PMC8568093 34735422

[25] Earp M, Mitchell M, McCarthy J, Kreuter F. Modeling nonresponse in establishment surveys: using an ensemble tree model to create nonresponse propensity scores and detect potential bias in an agricultural survey. J Off Stat 2014;30(4):701–19. 10.2478/JOS-2014-0044.

[26] Phipps P, Toth D. Analyzing establishment nonresponse using an interpretable regression tree model with linked administrative data. Ann Appl Stat 2012;6(2):772–94.

[27] Polivka AE. Data watch: the redesigned current population survey. J Econ Perspect 1996;10(3):169–80. Available from, https://www.jstor.org/stable/41713473. accessed 8 September 2024.

[28] Toth D, Phipps P. Regression tree models for analyzing survey response. In Proceedings of the Government Statistics Section. 2014. p. 339–51 [American Statistical Association].

[29] Morgan JN, Sonquist JA. Problems in the analysis of survey data, and a proposal. J Am Stat Assoc 1963;58(302):415–34. Available from, https://www.tandfonline.com/doi/abs/10.1080/01621459.1963.10500855.

[30] Toth D A permutation test on complex sample data. J Surv Stat Methodol 2020;8(4):772–91. 10.1093/jssam/smz018.

[31] Lu PJ, Zhou T, Santibanez TA, Jain A, Black CL, Srivastav A, COVID-19 bivalent booster vaccination coverage and intent to receive booster vaccination among adolescents and adults - United States, November-December 2022. MMWR Morb Mortal Wkly Rep 2023;72(7):190–8. 10.15585/mmwr.mm7207a5. 36795677 PMC9949845

[32] Hudson A, Montelpare WJ. Predictors of vaccine hesitancy: implications for COVID-19 public health messaging. Int J Environ Res Public Health 2021;18(15):8054. 10.3390/ijerph18158054. 34360345 PMC8345367

[33] Saelee R, Zell E, Murthy BP, Castro-Roman P, Fast H, Meng L, Disparities in COVID-19 vaccination coverage between urban and rural counties—United States, December 14, 2020–January 31, 2022. Morb Mortal Wkly Rep 2022;71(9):335. 10.15585/mmwr.mm7109a2 PMC8893338 35239636

