# Supplementary material for: Using regression tree analysis to examine demographic and geographic characteristics of COVID-19 vaccination trends over time, United States, May 2021–April 2022, National Immunization Survey Adult COVID Module
Source: Vaccine. Author manuscript; Available in PMC 2025 Apr 25. (PMC12024164; doi:10.1016/j.vaccine.2024.126372)
Supplement: Supplemental Tables : A-1, A-2, and A-3 [file NIHMS2063482-supplement-Supplemental_Tables___A-1__A-2__and_A-3.pdf]

Supplemental Tables

Table A-1 COVID-19 Vaccine Initiation Model: Covariate Proportions by Node

	Node5 (n=97,302)		Node6 (n=134,601)		Node7 (n=331,456)		Node8 (n= 85,774)		Node9 (n= 137,296)		Total
	n	(%)	n	(%)	n	(%)	n	(%)	n	(%)	
Age (years)											
18-29	18430	(18.94)	66195	(49.18)	0	(0.00)	0	(0.00)	47385	(34.51)	132010
30-39	14333	(14.73)	68406	(50.82)	0	(0.00)	0	(0.00)	44405	(32.34)	127144
40-49	13697	(14.08)	0	(0.00)	63116	(19.04)	0	(0.00)	41466	(30.20)	118279
50-64	25668	(26.38)	0	(0.00)	131056	(39.54)	60887	(70.99)	0	(0.00)	217611
65+	14594	(15.00)	0	(0.00)	90632	(27.34)	17844	(20.80)	0	(0.00)	123070
75+	8872	(9.12)	0	(0.00)	40195	(12.13)	7043	(8.21)	0	(0.00)	56110
Not reported	1708	(1.76)	0	(0.00)	6457	(1.95)	0	(0.00)	4040	(2.94)	12205
Race/Ethnicity											
Hispanic	22743	(23.37)	18500	(13.74)	26690	(8.05)	7771	(9.06)	22431	(16.34)	98135
NH American Indian/Alaskan Native	1361	(1.40)	1257	(0.93)	3257	(0.98)	1183	(1.38)	1986	(1.45)	9044
NH Asian	6845	(7.03)	12122	(9.01)	11214	(3.38)	1874	(2.18)	6747	(4.91)	38802
NH Black	17365	(17.85)	10100	(7.50)	30681	(9.26)	12454	(14.52)	17745	(12.92)	88345
NH Pacific Islander/Native Hawaiian	1464	(1.50)	840	(0.62)	2155	(0.65)	852	(0.99)	1590	(1.16)	6901
NH White	40079	(41.19)	84532	(62.80)	239422	(72.23)	55433	(64.63)	75758	(55.18)	495224
NH Multiple Race	3913	(4.02)	5526	(4.11)	8919	(2.69)	3104	(3.62)	6365	(4.64)	27827
Not reported	3532	(3.63)	1724	(1.28)	9118	(2.75)	3103	(3.62)	4674	(3.40)	22151
Sex											
Female	48691	(50.04)	73582	(54.67)	182412	(55.03)	38085	(44.40)	58950	(42.94)	401720
Male	47665	(48.99)	60394	(44.87)	147063	(44.37)	47311	(55.16)	76486	(55.71)	378919
Not reported	946	(0.97)	625	(0.46)	1981	(0.60)	378	(0.44)	1860	(1.35)	5790
Income											
Below poverty	16129	(16.58)	10854	(8.06)	20991	(6.33)	7776	(9.07)	15016	(10.94)	70766
LT 75K but above poverty	31253	(32.12)	44124	(32.78)	92758	(27.99)	28186	(32.86)	48006	(34.97)	244327
GT 75k	23080	(23.72)	58297	(43.31)	145822	(43.99)	28828	(33.61)	43803	(31.90)	299830

Not reported	26840	(27.58)	21326	(15.84)	71885	(21.69)	20984	(24.46)	30471	(22.19)	171506
Education											
GT high school	60611	(62.29)	109666	(81.47)	257787	(77.77)	55467	(64.67)	88830	(64.70)	572361
LTE high school	32429	(33.33)	23318	(17.32)	64974	(19.60)	27240	(31.76)	43979	(32.03)	191940
Not reported	4262	(4.38)	1617	(1.20)	8695	(2.62)	3067	(3.58)	4487	(3.27)	22128
Insurance											
Insured	82308	(84.59)	124085	(92.19)	315335	(95.14)	74642	(87.02)	109178	(79.52)	705548
Not insured	10661	(10.96)	8520	(6.33)	8481	(2.56)	8295	(9.67)	23208	(16.90)	59165
Not reported	4333	(4.45)	1996	(1.48)	7640	(2.30)	2837	(3.31)	4910	(3.58)	21716
Essential Worker (EW) status											
Healthcare	8821	(9.07)	24821	(18.44)	37085	(11.19)	4247	(4.95)	8039	(5.86)	83013
School/Childcare	3227	(3.32)	6388	(4.75)	13097	(3.95)	2719	(3.17)	4732	(3.45)	30163
Other frontline worker	5393	(5.54)	9045	(6.72)	14959	(4.51)	6759	(7.88)	15666	(11.41)	51822
Other EW	9671	(9.94)	15785	(11.73)	25821	(7.79)	11544	(13.46)	23433	(17.07)	86254
Not EW	69345	(71.27)	78067	(58.00)	239342	(72.21)	59879	(69.81)	84014	(61.19)	530647
Not reported	845	(0.87)	495	(0.37)	1152	(0.35)	626	(0.73)	1412	(1.03)	4530
Health Care Provider recommends vaccination											
No	53548	(55.03)	66879	(49.69)	158884	(47.94)	56684	(66.09)	96173	(70.05)	432168
Yes	42651	(43.83)	66486	(49.39)	168579	(50.86)	28380	(33.09)	40157	(29.25)	346253
Not reported	1103	(1.13)	1236	(0.92)	3993	(1.20)	710	(0.83)	966	(0.70)	8008
Work/School requirements											
No Work/School required	0	(0.00)	82031	(60.94)	198121	(59.77)	85774	(100.00)	137296	(100.00)	503222
Work/School required	57379	(58.97)	48679	(36.17)	58475	(17.64)	0	(0.00)	0	(0.00)	164533
Not reported	39923	(41.03)	3891	(2.89)	74860	(22.59)	0	(0.00)	0	(0.00)	118674
HHS Region[1]											
HHS1	8609	(8.85)	16023	(11.90)	43076	(13.00)	8560	(9.98)	11853	(8.63)	88121
HHS2	19694	(20.24)	10682	(7.94)	23856	(7.20)	8084	(9.42)	11160	(8.13)	73476
HHS3	14803	(15.21)	21326	(15.84)	51964	(15.68)	10590	(12.35)	17061	(12.43)	115744
HHS4	12000	(12.33)	15743	(11.70)	46602	(14.06)	14556	(16.97)	21931	(15.97)	110832
HHS5	7947	(8.17)	15452	(11.48)	35454	(10.70)	8433	(9.83)	14121	(10.29)	81407
HHS6	11605	(11.93)	16532	(12.28)	41472	(12.51)	11896	(13.87)	20684	(15.07)	102189

HHS7	2786	(2.86)	6642	(4.93)	16548	(4.99)	3743	(4.36)	6472	(4.71)	36191
HHS8	4607	(4.73)	10724	(7.97)	26763	(8.07)	7153	(8.34)	11653	(8.49)	60900
HHS9	10930	(11.23)	13289	(9.87)	28627	(8.64)	8225	(9.59)	13972	(10.18)	75043
HHS10	4321	(4.44)	8188	(6.08)	17094	(5.16)	4534	(5.29)	8389	(6.11)	42526
MSA status											
Non-MSA	16491	(16.95)	17632	(13.10)	63505	(19.16)	21669	(25.26)	27076	(19.72)	146373
MSA, non-principal city	44872	(46.12)	60410	(44.88)	166279	(50.17)	40868	(47.65)	64154	(46.73)	376583
MSA, principal City	35939	(18.47)	56559	(42.02)	101672	(30.67)	23237	(27.09)	46066	(33.55)	263473
Social Vulnerability Index (SVI)											
High	25918	(26.64)	33717	(25.05)	76660	(23.13)	21985	(25.63)	37045	(26.98)	195325
Medium	29270	(30.08)	47634	(35.39)	110956	(33.48)	27140	(31.64)	44731	(32.58)	259731
Low	20556	(21.13)	42418	(31.51)	110814	(33.43)	23617	(27.53)	36912	(26.88)	234317
Not reported	21558	(22.16)	10832	(8.05)	33026	(9.96)	13032	(15.19)	18608	(13.55)	97056
Previously had COVID											
No	73891	(75.94)	99221	(73.71)	268786	(81.09)	64533	(75.24)	95246	(69.37)	601677
Yes	22052	(22.66)	34294	(25.48)	59194	(17.86)	19695	(22.96)	40007	(29.14)	175242
Not reported	1359	(1.40)	1086	(0.81)	3476	(1.05)	1546	(1.80)	2043	(1.49)	9510
Other vaccine received											
No	95703	(98.36)	0	(0.00)	0	(0.00)	85093	(99.21)	135562	(98.74)	316358
Yes	0	(0.00)	134601	(100.00)	331456	(100.00)	0	(0.00)	0	(0.00)	466057
Not reported	1599	(1.64)	0	(0.00)	0	(0.00)	681	(0.79)	1734	(1.26)	4014

Abbreviation: NH-non-Hispanic; LT-less than; GT-greater than; GTE- greater than or equal to; HCP-Healthcare personnel; MSA- metropolitan statistical area

Footnote 1: The Office of Intergovernmental and External Affairs hosts 10 Health and Human Services (HHS) Regional Offices that directly serve state and local organizations. For details about HHS Regions: https://www.hhs.gov/about/agencies/regional-offices/index.html

Table A-2. COVID-19 Primary Series Completion Model: Covariate Proportions by Node

	Node8 (n=107,882)		Node9 (n=67,185)		Node20 (n=99,331)		Node21 (n=174,155)		Node11 (n=79,531)		Node3 (n=141,371)		Total
	n	(%)	n	(%)	n	(%)	n	(%)	n	(%)	n	(%)	
Age (years)													
18-29	0	(0.00)	30417	(45.27)	12347	(12.43)	12559	(7.21)	18547	(23.32)	28364	(20.06)	102234
30-39	29001	(26.88)	0	(0.00)	10068	(10.14)	22486	(12.91)	16124	(20.27)	24997	(17.68)	102676
40-49	28557	(26.47)	0	(0.00)	8098	(8.15)	24147	(13.87)	13587	(17.08)	23283	(16.47)	97672
50-64	50324	(46.65)	0	(0.00)	25300	(25.47)	51893	(29.80)	22251	(27.98)	40025	(28.31)	189793
65+	0	(0.00)	22290	(33.18)	28556	(28.75)	40429	(23.21)	6621	(8.33)	16778	(11.87)	114674
75+	0	(0.00)	11563	(17.21)	14429	(14.53)	19141	(10.99)	1355	(1.70)	6198	(4.38)	52686
Not reported	0	(0.00)	2915	(4.34)	533	(0.54)	3500	(2.01)	1046	(1.32)	1726	(1.22)	9720
Race/Ethnicity													
Hispanic	19582	(18.15)	11185	(16.65)	10685	(10.76)	11314	(6.50)	9814	(12.34)	20834	(14.74)	83414
NH American Indian/Alaskan Native	1265	(1.17)	664	(0.99)	1176	(1.18)	1077	(0.62)	643	(0.81)	1774	(1.25)	6599
NH Asian	6791	(6.29)	4778	(7.11)	3175	(3.20)	8435	(4.84)	8138	(10.23)	5726	(4.05)	37043
NH Black	17995	(16.68)	9985	(14.86)	10047	(10.11)	11822	(6.79)	8522	(10.72)	14997	(10.61)	73368
NH Pacific Islander/Native Hawaiian	1480	(1.37)	801	(1.19)	749	(0.75)	944	(0.54)	627	(0.79)	866	(0.61)	5467
NH White	54306	(50.34)	34205	(50.91)	69402	(69.87)	130357	(74.85)	47482	(59.70)	89252	(63.13)	425004
NH Multiple Race	3687	(3.42)	2576	(3.83)	3467	(3.49)	4246	(2.44)	2693	(3.39)	4524	(3.20)	21193
Not reported	2776	(2.57)	2991	(4.45)	630	(0.63)	5960	(3.42)	1612	(2.03)	3398	(2.40)	17367
Sex													
Female	51210	(47.47)	30084	(44.78)	57331	(57.72)	91016	(52.26)	44752	(56.27)	74536	(52.72)	348929
Male	56329	(52.21)	36063	(53.68)	41761	(42.04)	81969	(47.07)	34328	(43.16)	65893	(46.61)	316343
Not reported	343	(0.32)	1038	(1.54)	239	(0.24)	1170	(0.67)	451	(0.57)	942	(0.67)	4183
Income													
Below poverty	12068	(11.19)	8359	(12.44)	17648	(17.77)	0	(0.00)	4362	(5.48)	11113	(7.86)	53550

LT 75k but above poverty	34120	(31.63)	24113	(35.89)	81683	(82.23)	0	(0.00)	20302	(25.53)	44084	(31.18)	204302
GT 75k	39262	(36.39)	14440	(21.49)	0	(0.00)	116845	(67.09)	40961	(51.50)	57758	(40.86)	269266
Not reported	22432	(20.79)	20273	(30.17)	0	(0.00)	57310	(32.91)	13906	(17.49)	28416	(20.10)	142337
Education													
GT high school	75567	(70.05)	42185	(62.79)	70299	(70.77)	144810	(83.15)	67949	(85.44)	106364	(75.24)	507174
LTE high school	29282	(27.14)	21607	(32.16)	28733	(28.93)	23372	(13.42)	10084	(12.68)	31535	(22.31)	144613
Not reported	3033	(2.81)	3393	(5.05)	299	(0.30)	5973	(3.43)	1498	(1.88)	3472	(2.46)	17668
Insurance status													
Insured	91666	(84.97)	57661	(85.82)	94784	(95.42)	164905	(94.69)	75156	(94.50)	127807	(90.41)	611979
Not Insured	13273	(12.30)	6114	(9.10)	4385	(4.41)	3680	(2.11)	2937	(3.69)	10099	(7.14)	40488
Not reported	2943	(2.73)	3410	(5.08)	162	(0.16)	5570	(3.20)	1438	(1.81)	3465	(2.45)	16988
Essential Worker (EW) status													
Healthcare	8883	(8.23)	2911	(4.33)	6479	(6.52)	14590	(8.38)	23146	(29.10)	19447	(13.76)	75456
School/Childcare	5204	(4.82)	1468	(2.19)	3190	(3.21)	7239	(4.16)	4199	(5.28)	6656	(4.71)	27956
Other frontline worker	9648	(8.94)	3732	(5.55)	4618	(4.65)	8022	(4.61)	3825	(4.81)	10251	(7.25)	40096
Other EW	15683	(14.54)	5775	(8.60)	6618	(6.66)	13939	(8.00)	8505	(10.69)	16515	(11.68)	67035
Not EW	67878	(62.92)	52852	(78.67)	78217	(78.74)	129819	(74.54)	39490	(49.65)	87871	(62.16)	456127
Not reported	586	(0.54)	447	(0.67)	209	(0.21)	546	(0.31)	366	(0.46)	631	(0.45)	2785
Health Care Provider recommends vaccination													
No	67471	(62.54)	43308	(64.46)	51173	(51.52)	88534	(50.84)	33618	(42.27)	67161	(47.51)	351265
Yes	39595	(36.70)	23193	(34.52)	47081	(47.40)	83247	(47.80)	45095	(56.70)	72889	(51.56)	311100
Not reported	816	(0.76)	684	(1.02)	1077	(1.08)	2374	(1.36)	818	(1.03)	1321	(0.93)	7090
Work/School requirements													
No Work/School required	73609	(68.23)	37827	(56.30)	68880	(69.34)	140537	(80.70)	0	(0.00)	84774	(59.97)	405627
Work/School required	24928	(23.11)	13350	(19.87)	0	(0.00)	0	(0.00)	79531	(100.00)	40513	(28.66)	158322
Not reported	9345	(8.66)	16008	(23.83)	30451	(30.66)	33618	(19.30)	0	(0.00)	16084	(11.38)	105506
HHS Region[1]													
HHS1	11162	(10.35)	6524	(9.71)		(12.24)	23846	(13.69)		(15.05)	14221	(10.06)	79879

					12155				11971				
HHS2	16347	(15.15)	9721	(14.47)	7045	(7.09)	11229	(6.45)	8682	(10.92)	12421	(8.79)	65445
HHS3	16358	(15.16)	8861	(13.19)	12625	(12.71)	29135	(16.73)	16756	(21.07)	18854	(13.34)	102589
HHS4	14432	(13.38)	9268	(13.79)	14483	(14.58)	23435	(13.46)	6952	(8.74)	21227	(15.02)	89797
HHS5	9781	(9.07)	6121	(9.11)	10784	(10.86)	19099	(10.97)	8123	(10.21)	15763	(11.15)	69671
HHS6	13600	(12.61)	8754	(13.03)	13874	(13.97)	20768	(11.93)	7554	(9.50)	19444	(13.75)	83994
HHS7	3514	(3.26)	2475	(3.68)	5337	(5.37)	8305	(4.77)	2592	(3.26)	7506	(5.31)	29729
HHS8	5593	(5.18)	4263	(6.35)	8630	(8.69)	13066	(7.50)	3950	(4.97)	12878	(9.11)	48380
HHS9	11732	(10.87)	7756	(11.54)	8654	(8.71)	15839	(9.09)	8642	(10.87)	12116	(8.57)	64739
HHS10	5363	(4.97)	3442	(5.12)	5744	(5.78)	9433	(5.42)	4309	(5.42)	6941	(4.91)	35232
MSA status													
Non-MSA	18947	(17.56)	12964	(19.30)	22182	(22.33)	28310	(16.26)	8656	(10.88)	23764	(16.81)	114823
MSA, Non-principal city	52183	(48.37)	30667	(45.65)	45300	(45.61)	90465	(51.95)	37258	(46.85)	66685	(47.17)	322558
MSA, principal city	36752	(34.07)	23554	(35.06)	31849	(32.06)	55380	(31.80)	33617	(42.27)	50922	(36.02)	232074
Social Vulnerability Index (SVI)													
High	27447	(25.44)	16588	(24.69)	27175	(27.36)	34708	(19.93)	18354	(23.08)	38908	(27.52)	163180
Medium	34198	(31.70)	20731	(30.86)	34545	(34.78)	57927	(33.26)	27834	(35.00)	47239	(33.41)	222474
Low	28298	(26.23)	16208	(24.12)	30978	(31.19)	61709	(35.43)	25913	(32.58)	41635	(29.45)	204741
Not reported	17939	(16.63)	13658	(20.33)	6633	(6.68)	19811	(11.38)	7430	(9.34)	13589	(9.61)	79060
Previously had COVID													
No	107882	(100.00)	67185	(100.00)	99331	(100.00)	174155	(100.00)	79531	(100.00)	0	(0.00)	528084
Yes	0	(0.00)	0	(0.00)	0	(0.00)	0	(0.00)	0	(0.00)	134515	(95.15)	134515
Not reported	0	(0.00)	0	(0.00)	0	(0.00)	0	(0.00)	0	(0.00)	6856	(4.85)	6856
Other vaccine received													
No	107882	(100.00)	67185	(100.00)	0	(0.00)	0	(0.00)	0	(0.00)	53393	(37.77)	228460
Yes	0	(0.00)	0	(0.00)	98564	(99.23)	172926	(99.29)	78968	(99.29)	87239	(61.71)	437697
Not reported	0	(0.00)	0	(0.00)	767	(0.77)	1229	(0.71)	563	(0.71)	739	(0.52)	3298

Abbreviation: NH-non-Hispanic; LT-less than; GT-greater than; GTE- greater than or equal to; HCP-Healthcare personnel; MSA- metropolitan statistical area

Footnote 1: The Office of Intergovernmental and External Affairs hosts 10 Health and Human Services (HHS) Regional Offices that directly serve state and local organizations. For details about HHS Regions: https://www.hhs.gov/about/agencies/regional-offices/index.html

Table A-3 First COVID-19 Booster Dose Completion Model: Covariate Proportions by Node

	Node4 (n=64,892)		Node5 (n=57,124)		Node6 (n=98,983)		Node7 (n=144,505)		Total
	n	(%)	n	(%)	n	(%)	n	(%)	
Age (years)									
18-29	23815	(36.70)	0	(0.00)	31990	(32.32)	0	(0.00)	55805
30-39	20762	(31.99)	0	(0.00)	34853	(35.21)	0	(0.00)	55615
40-49	20315	(31.31)	0	(0.00)	32140	(32.47)	0	(0.00)	52455
50-64	0	(0.00)	34445	(60.30)	0	(0.00)	68480	(47.39)	102925
65+	0	(0.00)	13958	(24.43)	0	(0.00)	49959	(34.57)	63917
75+	0	(0.00)	6811	(11.92)	0	(0.00)	22798	(15.78)	29609
Not reported	0	(0.00)	1910	(3.34)	0	(0.00)	3268	(2.26)	5178
Race/Ethnicity									
Hispanic	14040	(21.64)	8387	(14.68)	12740	(12.87)	10417	(7.21)	45584
NH American Indian/Alaskan Native	704	(1.08)	654	(1.14)	844	(0.85)	1259	(0.87)	3461
NH Asian	5626	(8.67)	1563	(2.74)	8510	(8.60)	3829	(2.65)	19528
NH Black	8763	(13.50)	9686	(16.96)	7556	(7.63)	12989	(8.99)	38994
NH Pacific Islander/Native Hawaiian	639	(0.98)	439	(0.77)	460	(0.46)	631	(0.44)	2169
NH White	31405	(48.40)	32101	(56.20)	64225	(64.88)	107769	(74.58)	235500
NH Multiple Race	2485	(3.83)	1710	(2.99)	3478	(3.51)	3379	(2.34)	11052
Not reported	1230	(1.90)	2584	(4.52)	1170	(1.18)	4232	(2.93)	9216
Sex									
Female	29926	(46.12)	27469	(48.09)	56480	(57.06)	78101	(54.05)	191976
Male	34641	(53.38)	29003	(50.77)	42131	(42.56)	65519	(45.34)	171294
Not reported	325	(0.50)	652	(1.14)	372	(0.38)	885	(0.61)	2234
Income									
Below poverty	7017	(10.81)	6299	(11.03)	6343	(6.41)	8534	(5.91)	28193
LT 75K but above poverty	22516	(34.70)	18266	(31.98)	27862	(28.15)	42706	(29.55)	111350
GT 75K	21868	(33.70)	16811	(29.43)	48897	(49.40)	60518	(41.88)	148094
Not reported	13491	(20.79)	15748	(27.57)	15881	(16.04)	32747	(22.66)	77867
Education									
GT high school	46447	(71.58)	36329	(63.60)	83960	(84.82)	111086	(76.87)	277822

LTE high school	17058	(26.29)	18057	(31.61)	13937	(14.08)	29290	(20.27)	78342
Not reported	1387	(2.14)	2738	(4.79)	1086	(1.10)	4129	(2.86)	9340
Insurance									
Insured	54086	(83.35)	50305	(88.06)	92857	(93.81)	138034	(95.52)	335282
Not insured	9152	(14.10)	4327	(7.57)	4900	(4.95)	2824	(1.95)	21203
Not reported	1654	(2.55)	2492	(4.36)	1226	(1.24)	3647	(2.52)	9019
Essential Worker (EW) status									
Healthcare	5301	(8.17)	3323	(5.82)	19323	(19.52)	13425	(9.29)	41372
School/Childcare	2896	(4.46)	1876	(3.28)	5912	(5.97)	4966	(3.44)	15650
Other frontline worker	5945	(9.16)	3358	(5.88)	5720	(5.78)	5443	(3.77)	20466
Other EW	9108	(14.04)	6017	(10.53)	10757	(10.87)	9612	(6.65)	35494
Not EW	41293	(63.63)	42200	(73.87)	56992	(57.58)	110643	(76.57)	251128
Not reported	349	(0.54)	350	(0.61)	279	(0.28)	416	(0.29)	1394
Health Care Provider recommends vaccination									
No	39586	(61.00)	33592	(58.81)	43023	(43.47)	66619	(46.10)	182820
Yes	24789	(38.20)	22973	(40.22)	54960	(55.52)	75971	(52.57)	178693
Not reported	517	(0.80)	559	(0.98)	1000	(1.01)	1915	(1.33)	3991
Work/School requirements									
No Work/School required	38274	(58.98)	32215	(56.39)	48899	(49.40)	75939	(52.55)	195327
Work/School required	24229	(37.34)	11310	(19.80)	47135	(47.62)	28754	(19.90)	111428
Not reported	2389	(3.68)	13599	(23.81)	2949	(2.98)	39812	(27.55)	58749
HHS Region[1]									
HHS1	6495	(10.01)	5889	(10.31)	12827	(12.96)	20490	(14.18)	45701
HHS2	9027	(13.91)	8689	(15.21)	7985	(8.07)	10807	(7.48)	36508
HHS3	8405	(12.95)	7119	(12.46)	15331	(15.49)	20271	(14.03)	51126
HHS4	8539	(13.16)	9051	(15.84)	11351	(11.47)	20536	(14.21)	49477
HHS5	6624	(10.21)	5590	(9.79)	11984	(12.11)	16497	(11.42)	40695
HHS6	8494	(13.09)	7232	(12.66)	11686	(11.81)	17420	(12.05)	44832
HHS7	2474	(3.81)	2147	(3.76)	5270	(5.32)	7270	(5.03)	17161
HHS8	4275	(6.59)	3805	(6.66)	8102	(8.19)	12567	(8.70)	28749
HHS9	7166	(11.04)	5254	(9.20)	8817	(8.91)	12205	(8.45)	33442

HHS10	3393	(5.23)	2348	(4.11)	5630	(5.69)	6442	(4.46)	17813
MSA status									
Non-MSA	8970	(13.82)	12010	(21.02)	12261	(12.39)	28163	(19.49)	61404
MSA, Non-principal city	29162	(44.94)	26748	(46.82)	44023	(44.48)	70482	(48.77)	170415
MSA, principal city	26760	(41.24)	18366	(32.15)	42699	(43.14)	45860	(31.74)	133685
Social Vulnerability Index (SVI)									
High	17663	(27.22)	15024	(26.30)	24551	(24.80)	33787	(23.38)	91025
Medium	21349	(32.90)	16940	(29.65)	34845	(35.20)	47853	(33.12)	120987
Low	17201	(26.51)	14175	(24.81)	33708	(34.05)	49667	(34.37)	114751
Not reported	8679	(13.37)	10985	(19.23)	5879	(5.94)	13198	(9.13)	38741
Previously had COVID									
No	43150	(66.50)	43453	(76.07)	68830	(69.54)	115046	(79.61)	270479
Yes	21036	(32.42)	12858	(22.51)	29406	(29.71)	28052	(19.41)	91352
Not reported	706	(1.09)	813	(1.42)	747	(0.75)	1407	(0.97)	3673
Other vaccine received									
No	63915	(98.49)	56508	(98.92)	0	(0.00)	0	(0.00)	120423
Yes	0	(0.00)	0	(0.00)	98983	(100.00)	144505	(100.00)	243488
Not reported	977	(1.51)	616	(1.08)	0	(0.00)	0	(0.00)	1593

Abbreviation: NH-non-Hispanic; LT-less than; GT-greater than; GTE- greater than or equal to; HCP-Healthcare personnel; MSA- metropolitan statistical area

Footnote 1: The Office of Intergovernmental and External Affairs hosts 10 Health and Human Services (HHS) Regional Offices that directly serve state and local organizations. For details about HHS Regions: https://www.hhs.gov/about/agencies/regional-offices/index.html
